# Ambient Assisted Living: A Review of Technologies, Methodologies and Future Perspectives for Healthy Aging of Population

**DOI:** 10.3390/s21103549

**Published:** 2021-05-19

**Authors:** Grazia Cicirelli, Roberto Marani, Antonio Petitti, Annalisa Milella, Tiziana D’Orazio

**Affiliations:** Institute of Intelligent Industrial Systems and Technologies for Advanced Manufacturing (STIIMA), National Research Council of Italy (CNR), Via G. Amendola 122, 70126 Bari, Italy; roberto.marani@stiima.cnr.it (R.M.); antonio.petitti@stiima.cnr.it (A.P.); annalisa.milella@stiima.cnr.it (A.M.); tiziana.dorazio@stiima.cnr.it (T.D.)

**Keywords:** active assisted living, wearable sensors, smart objects, environmental sensors, data collection, methodologies for data analysis

## Abstract

Over the last decade, there has been considerable and increasing interest in the development of Active and Assisted Living (AAL) systems to support independent living. The demographic change towards an aging population has introduced new challenges to today’s society from both an economic and societal standpoint. AAL can provide an arrary of solutions for improving the quality of life of individuals, for allowing people to live healthier and independently for longer, for helping people with disabilities, and for supporting caregivers and medical staff. A vast amount of literature exists on this topic, so this paper aims to provide a survey of the research and skills related to AAL systems. A comprehensive analysis is presented that addresses the main trends towards the development of AAL systems both from technological and methodological points of view and highlights the main issues that are worthy of further investigation.

## 1. Introduction

In the last decade, smart environments have found wide application in several contexts, such as domotic, education, rehabilitation, assistance, and so on. Structuring classroom environments with new technologies makes learning more attractive, as well as providing support for teaching people with disabilities [1]. Similarly, new technological devices provide more specific tools and support for the rehabilitation of people with impairment [2,3]. In the context of home automation, the deployment of new technologies can improve the quality of daily life, provide easy access to many functions remotely, or allow the use of natural interfaces, such as gestures and voice, in order to control lighting, climate, entertainment systems, and appliances [4,5].

Contextually, a new need has recently emerged: as the population ages, new policies, systems, and technologies for supporting healthy aging are needed [6]. Aging leads to gradual physical decay, which increases the dependency on other people. Starting from this consideration, two different aspects can be examined. On the one hand, new technologies can be used to monitor older people in order to reduce daily physical assistance and prolong their autonomous life. In this context, medicine and diet adherence, sleep monitoring, or fall detection are some of the main issues that are to be considered and tackled. On the other hand, monitoring people who are not yet very old and that do not suffer from particular pathologies is strategic for the purpose of detecting eventual lifestyle changes and suspicious behaviors that could warn of the onset of neurodegenerative diseases at a very early stage.

In gerontology literature, the decrease in physical activity is considered to be strictly correlated with ageing, and it is associated with low social function, depression, and cognitive decline [7]. A focus on physical, social and cognitive activity is needed in order to promote active and healthy ageing, and to permit people to live independently for longer [8]. Active Assisted Living (AAL) technologies can intervene to help elderly people in the different stages of aging. Indeed, AAL is intended to develop innovation in order to keep people connected, healthy, active, and happy into their old age. It concerns the development of products and services that make a real difference to people’s lives, for those who face some of the challenges of ageing and those who care for older people who need help.

In recent years, a growing body of literature has examined the state of the art of AAL domain by different points of view. In [9,10], the authors focused on the technologies for Activity Recognition in Smart Home. In [11], a review of the literature on mobile technology, such as smartphones, smartwatches, and wristbands, is presented, which describes how these wearable technologies can be used to promote an active lifestyle. Non-wearable (i.e., ambient) technologies are surveyed in [12] for the development of various elderly care systems, whereas IoT Wearable Sensors are reviewed in [13,14]. In [15], video-based technologies have been reviewed. The AAL system requirements and implementation challenges examining the existing Reference Models and Reference Architectures, from an architectural point of view, are investigated in [16], which highlight both the quality attributes and critical issues.

This paper surveys the recent literature on AAL systems from a different perspective, with the aim of providing an exhaustive exploration of contexts, technologies, and existing approaches to figure out the actual needs for developing a complete AAL system. In recent years, as the great technological progress has led to the spread on the market of several kinds of sensors, many papers and research projects have been presented. In this paper, a selection of the more recent published works has been discussed while considering the application contexts, the potential users, and the functionalities of the proposed systems, as well as the technologies and methodologies that are used for their development. The referenced papers have been selected by searching the main bibliographic databases, including IEEE Xplore, ScienceDirect, ACM, and open access journals, and considering those that were published in the previous five years. During the search, some key terms were used, such as “ambient assisted living technology”, “elderly”, “daily activities”, “smart home”, “ambient sensors”, and “activity monitoring”, with both AND and OR connectives. The screening method included studies that more closely met a predefined set of characteristics that are useful for contributing significantly to the discussion carried out in the paper.

The application context, such as indoor/outdoor environments or private/public environments, imposes strict constraints on the type of technologies to be used; invasive or not, with short or long range distance, and so on. Indeed, the use of different sensors can greatly change the parameters that can be observed. In addition, the category of people to be monitored certainly influences the choice of the technology: people with diseases, people with disabilities, healthy people, and so on. The choice of the methodology for data processing is undoubtedly an additional fundamental aspect involved in the development of AAL systems. There exist several methodologies in the literature, having different peculiarities depending on the complexity of the AAL system output. This, in fact, can vary from a simple alarm, alerting when parameters are out of range, to the classification of complex behaviors that were carried out from the observation and correlation of the temporal sequences of parameters. The aim of this review is to provide an overview of the main issues that are related to the development of a complete AAL system, to highlight the choices that need to be made in terms of sensors and methodologies, in order to realize systems that implement different functionalities, with different target users, and in different possible environments.

The rest of this paper is organized, as follows. In Section 2, different application contexts are considered that focused on the main requirements needed for parameter observation. Section 3 illustrates a panoramic survey of the different sensory technologies. Section 4 presents the methodologies for data analysis and decision making. Finally, a critical discussion is provided in Section 5 to determine the main points to address in future research.

## 2. Application Contexts

**Figure 1:**
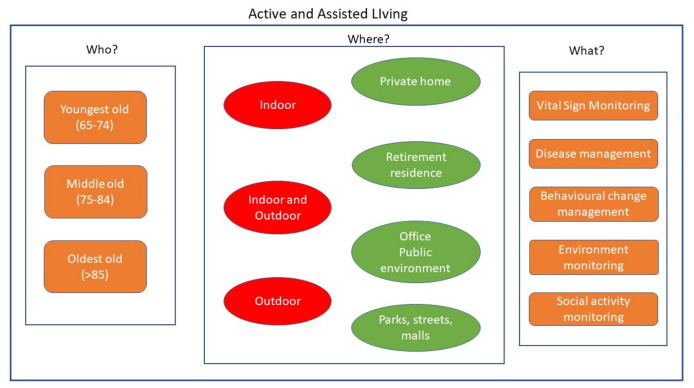
Fundamental aspects to be considered in the design and development of an AAL system: possible users (who?), environments (where?), and functionalities (what?).

The design and development of an AAL system involve different aspects that can be summarized in three principal words: who, where, and what. Target people are persons with different abilities and skills that have to be monitored by the system (*who*). The application contexts vary from indoor to outdoor environments and, hence, involve different solutions from both the technological and methodological points of view (*where*). Finally, an AAL system implies a wide range of functionalities that span from simple alerts when dangerous events happen, to more complex behavioral analysis (*what*). Figure 1 outlines these aspects in a sketch and they are described in the following sections.

### 2.1. Target Users

AAL systems are principally targeted to elderly adults, aiming maintain continuous support and prolong their autonomous life in an active and healthy way. Medical studies have categorized elderly people into three different classes, depending on their age: those between the ages of 65 and 74 years are classified as *youngest-old*, those between 75 and 84 years old as *middle-old*, and those aged over 85 years as *oldest-old* [17]. For all of these classes of people, health practices are aimed at disease management, at alarm alerting, or at behavioral change management. In recent years, several applications for diabetes control, depression treatment, hypertension control, medication adherence, and psychological support have been developed to allow people to live alone while having the possibility of daily control over their health status. Alarm alerting applications can save human life, when critical events, such as falls, prolonged inactivity, or environmental dangers, are detected. The early detection of behavioral changes is necessary before a notable deterioration of the basic activities involved in daily living [18]. Scientific studies have pointed out that the activity levels of people drop significantly when they retire. By the early identification of the risk factors of functional decline, this can be prevented in a large portion of the elderly population at the time of retirement by targeting timely interventions and reducing the risks. Furthermore, behavioral change applications include monitoring some dangerous attitudes, such as smoking, calorie intake for diet and exercise, and physical activity levels.

### 2.2. Indoor Environments

In the literature, most of the researches focus on AAL systems for *indoor environments*. One of the main application contexts related to medical and public health practices supported by devices that deliver health care services via mobile communication. New systems and methods have been developed for the continuous monitoring of biological, behavioral, or environmental data, delivering interventions, and assessing their outcomes. Through the development of systems collecting data coming from heterogeneous sensors and additional self-reported data, new information regarding physiological, psychological, emotional, and environmental states can be derived.

**Figure 2:**
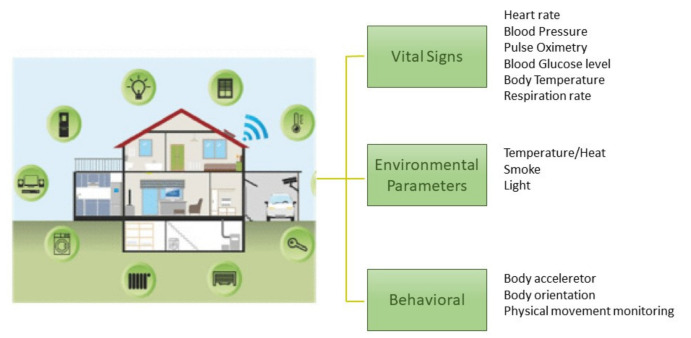
Several types of sensors deployed into the house and attached to devices permit gathering a variety of data concerning the location of the resident(s), the object(s) they communicate with, and data related to health conditions (from [19]).

**Figure 3:**
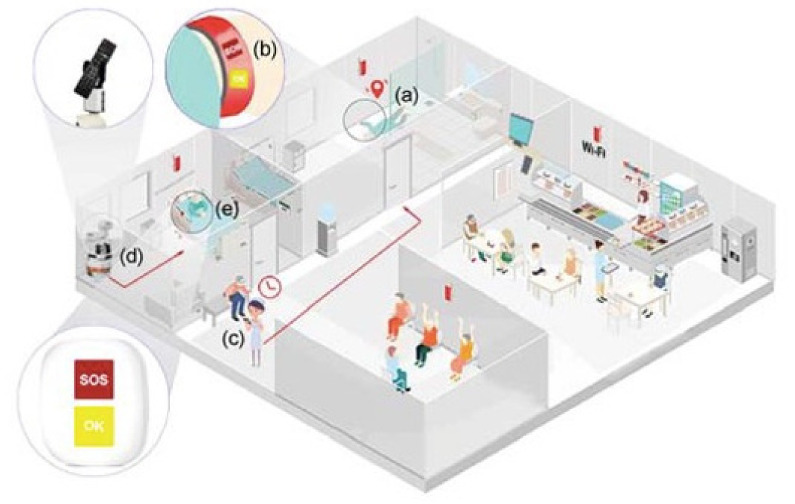
A retirement home (from [20]): heterogeneous sensors can collect data to support the caregivers in their work, while, at the same time, collect valuable data for the indoor localization of both residents and caregivers. The system detects a fallen person through the signal sent by the (**a**,**b**) Wi-fi bracelet and alerts (**c**) the caregiver. The (**d**) robot assists a (**e**) bedridden resident requesting help.

Indoor environments can be further differentiated in *homes* [19], where people live alone or with a few relatives (see Figure 2) and *retirement residences*, where more people live together, move in common spaces, perform group or individual activities, and undertake controlled physical activities [20] (see Figure 3). Health status evaluation can be carried out by observing people movements, recognizing their actions, evaluating resting periods, monitoring food intake, and so on. Behavioral analysis can be done by detecting anomalies while comparing the actual behavior with the expected one [21]. Social activities in a group or interactions with relatives and friends can also be monitored.

### 2.3. Outdoor Environments

Elderly people spend a number of hours of the day outside the home environment. Recent studies have pointed out the need for seniors to spend time in outdoor environments, as they are motivated to be more active not only physically, but also spiritually and socially [22]. Moreover, outdoor habits, such as walking, shopping, meeting other people, and performing physical activities, can greatly help in the prevention of functional decline. If, on one hand, this is widely recognized to be very beneficial for elderly people, on the other hand it inevitably causes new safety concerns. In outdoor environments, elderly people can be exposed to several risks, such as falls or excessive heat or cold. Furthermore, in the case of people having early symptoms of dementia, wandering and becoming confused or lost are common risks. In these scenarios, the main objective of AAL systems is to provide support to elderly people in various aspects, such as in checking the routes, recognizing anomalous behaviors, evaluating motion activities, and so on.

**Figure 4:**
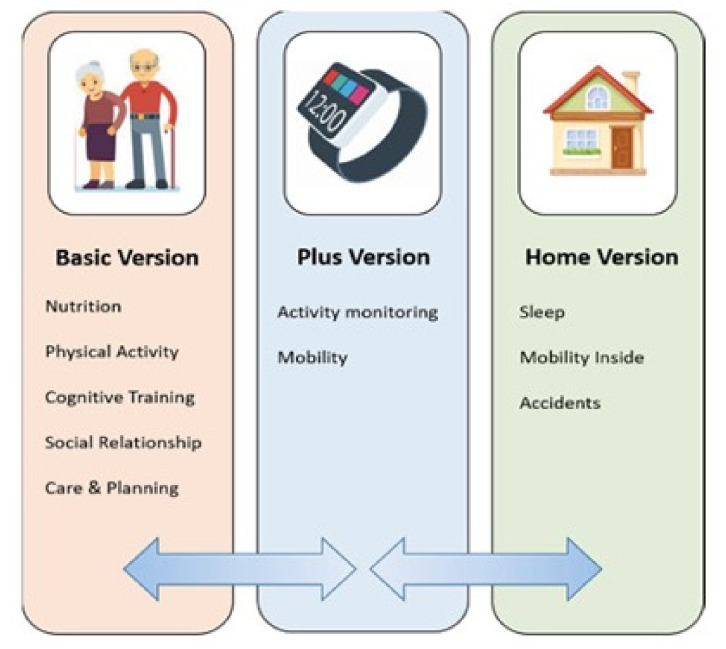
The solution proposed in [26] for activity monitoring in multiple domains of preventive measures: nutritional guidance, physical exercise promotion, cognitive practice, social activity, and positive care planning.

In recent years, several projects have developed different solutions to expand ambient assisted systems from indoor spaces to outdoor and public environments, with the aim of creating different services for improving the wellbeing of people [23]. In general, these projects gather data from people observations, store them securely, and, by their analysis, create intervention systems that can be applied for both general or specific monitoring scenarios. These systems can support elderly people by giving them hints and suggestions that are based on the analyzed data acquired daily [24,25] in order to slow the progression of their cognitive and behavioral decline (see Figure 4) [26].

## 3. Technologies

In accordance with the application domains, different technologies can be used for developing AAL systems, starting from simple IoT devices, to more complex sensor networks composed by environmental sensors, intelligent devices, video cameras, and so on. The variety of technologies automatically implies a greater complexity of data, as they can change considerably in terms of size, heterogeneity, and sampling frequency. Data management involves several issues, such as communication protocols, security controls, energy consumption, failure detection, interoperability among multi vendor devices, and so on. The aspects concerning privacy and the protection of sensitive data must consider the legal obligations that may arise whenever a system provides for the collection, storage, and transmission of data whose misuse can compromise people’s rights and freedoms. These technical issues are not discussed here, as they fall outside the scope of this review, but their significance is widely recognized for the development of complex AAL architectures, as extensively documented in literature [27,28,29,30,31,32,33,34].

This section examines the fundamental technologies that are used for people monitoring in AAL scenarios. Table 1 resumes the principal technologies applied in various contexts to create different health assistance tasks. The rapid progress of ICT technologies has recently made the discovery and deployment of many multi-functional devices possible. This has widely encouraged the development of effective AAL systems by the combination of ICT technologies and sensor technologies. Many devices, like smart-objects, connected sensors, wearable sensors, smartphones, and smartwatches, have been integrated with non-invasive sensors, such as cameras or infrared sensors, to build systems for people monitoring in both indoor and outdoor applications. As can be observed, the variety of technologies is wide enough to fulfill some particular constraints: to be non-invasive, to be easily acceptable by subjects, and to not affect users in their normal activities. Indeed, as reported above, the potential users of AAL systems are subjects with special needs and individuals having their own relational networks made up of relatives and friends and carrying out even complex activities, in the context of the home and city.

In the following, four principal categories of technologies will be examined. We start from those that can be easily used, such as wearable sensors, but that require user acceptance, and then move on to those that are less invasive, but that require structuring objects or furniture, i.e., smart everyday objects, up to environmental sensors and social assistive robots.

### 3.1. Wearable Sensors

In recent years, a number of wearable sensors have been miniaturized and made very efficient in terms of energy consumption. Three-axis accelerometers, gyroscopes, and magnetometers are the most commonly used wearable sensors, which are usually worn at the user hip or waist. They have been extensively applied for different purposes, such as to assess the postural stability of a subject [35], to detect and classify falls [36,37,38,39,40], or to analyze the gait cycle [41]. Moreover, these sensors are embedded in mobile technologies, such as smartphones, smartwatches, and wristbands, which also support the continuous monitoring of biological, behavioral, and environmental data [42,43,44].

Solutions that are based on passive Radio-Frequency Identification (RFID) technology have been largely used to identify the dynamic position of people moving in indoor environments. Information regarding human motion is carried out when wearable electromagnetic markers (tags) move within an electromagnetic field that is radiated by an interrogating antenna and sensed by the remote receiver or reader [45,46]. This type of system not only monitors movements and location, but also accidental falls [47].

An additional technology that has undergone significant developments over the last two decades is Bluetooth Low Energy (BLE) technology, which has paved the way to Bluetooth Smart for wearable devices, which has also solved the limitations related to low broadband connectivity in some particular environments. This technology has been considered for healthcare applications, such as daily physical activity monitoring, health monitoring in cardiac patients, intelligent stethoscope development, emotional level diagnosis [48], falls, and location of elderly people [49]. When multiple areas of an environment are equipped with BLE scanners and BLE beacons are attached to home residents, the system records residents’ activities, which helps to localize and track them [50], detect risky areas, and to prevent injuries and dangerous events without intruding on personal privacy [51].

The AAL systems examined so far, principally monitor the behavioral status of people. Additional relevant parameters have to be monitored in order to ascertain overall health status of an individual. These are the physiological parameters, which are also known as *vital signs*, such as heart rate (HR), blood pressure, body temperature, respiratory rate, and blood oxygen saturation. Keeping track of vital bodily functions can be done through different sensors that can be complementary to the aforementioned sensors in order to build complete long-term health monitoring systems in daily-life scenarios. Recent improvements in the performance and cost-effectiveness of microelectro-mechanical systems have opened new possibilities in continuous vital sign monitoring. In particular, ballistocardiography (BCG) and seismocardiography (SCG) are promising techniques for extracting information on cardiac events and phases. They are based on IMU sensors positioned over the subject’s sternum for assessing the breathing rate and quality metrics of physical activities [52], or for measuring vibrations that are produced by the heart’s mechanical activity [53]. Smart technology is also used to equip portable objects, such as glasses, in order to evaluate some vital signs, such as heart rate, respiratory rate, regularity of pulse and respiration, the presence and length of apnea events, the temperature distribution on the face, etc. [54]. Such methods are very important for healthcare professionals, because they allow for collecting important medical information without the connection of dedicated hardware to the patient, but with a more natural method.

In outdoor contexts, standard mobile devices can be used for increasing the elderly’s independence without them having to interact with the technology. By using the built-in sensors of cellphones, normal activities can be discerned from those that are considered not allowed and dangerous for the health of the observed individuals [55]. By integrating information that is provided by a GPS and accelerometer with online weather data, it is possible to infer physical and mental health information, such as long exposure to hazardous weather, wandering, or getting lost in order to create safe outdoor spaces [56]. Sensor boxes that are interfaced with mobile/phone applications acting as intelligent navigators can make street navigation accessible to impaired people [57].

### 3.2. Smart Everyday Objects

With the recent emergence of Internet of Things (IoT) and miniaturized sensors and processors, everyday objects can be identifiable, and they possess the ability to communicate and interact: in a word they can be smart. In this way, home appliances can communicate with each other or be controlled remotely, providing home residents with new facilities that are able to detect anomalies or assess health issues early, in order to apply prevention policies or trigger actions [58,59,60]. In AAL contexts, smart every day objects are generally used together with wearable sensors to acquire joint knowledge regarding individual activities and interactions with the objects in the environment [61,62,63].

Home cooking is one of the basic activities in a daily routine. Monitoring elderly people when they use kitchen appliances is very important in preventing injures and increasing safety. The first signs of cognitive disorders are often easily seen in behaving variations during meal preparation or cooker handling. Some common appliances, such as refrigerators, microwaves, and cooktops, are interconnected with each other to provide assistance in meal preparation tasks [64]. Furthermore, specific conditions, such as the presence of objects on cookers, the presence of lids on the pots, and the liquid level in the pots, can be monitored during the cooking process [65].

Sensors in furniture, such as doors, beds, chairs, washstand, toilet, and cupboards, allow for the non-obtrusive monitoring of daily living activities, providing presence statistics of users in different spaces of the environment [62,66]. Furthermore, power meters can be used to monitor appliance usage, such as TV set or lamps [63], whereas smart pill box devices can be very useful for checking medication intake [61].

Many other solutions have been proposed to provide intelligent functionalities to specific objects and monitor some specific issues that are related to the health and safety of elderly people. Cushion sensors, for example, can be used in wheelchairs to track user posture [67] or capture the heart rate of a person sitting on it [68]. Smart carpets and smart walls can have various functionalities, such as monitoring the walking activity of people, in order to detect falls, measure gait, and count the number of people traversing the environment (socializing activity) [69,70].

### 3.3. Environmental Sensors

Environmental monitoring sensors are used to detect parameters, such as temperature or air quality, which could have an adverse impact on elderly people [71,72]. They also contribute to monitoring daily life activities [73,74] or localizing people [75,76,77,78] and objects around people [79]. Unlike wearable sensors and smart object sensors, environmental sensors are placed in the environment without being invasive for people and without structuring or replacing home objects.

Radio-frequency-based systems, through the analysis of the reflections of radio-frequency signals, can be used for monitoring different people’s activities, such as sleep monitoring by inferring the subjects’ sleep postures [80], capturing people’s 3D dynamics, learning people’s interactions with the objects [81], and for detecting changes in movement patterns [82]. Microwave sensors can operate through optically opaque materials, such as clothing, as they are not impacted by external visible lighting and scene color, and they may eventually help to sense through fog and smoke. Intelligent metasurface systems transform measured microwave data into images and allow for the recognition of hand signs and vital signs of multiple non-cooperative people [83,84,85].

Intelligent optical systems are an example of environmental sensory systems that are used for biomedical signal acquisition to reveal rigid and uncontrollable gestures, postural instability, or small tremor, which can be signs of incoming neurological diseases. Low-cost devices, such as Kinect™and Wii™, can be easily installed for monitoring the daily activity of people in order to keep an active and healthy life under observation and to notify caregivers in the case of calls for help [2,86,87].

### 3.4. Social Assistive Robots

Social Assistive Robots are less common than wearable or environmental sensors, but they represent an emerging technology that can support and enhance human activities, especially in heavy tasks [88]. Social Assistive Robots can be used for different purposes: to overcome elderly inabilities; to relieve nursing staff from time-consuming, non-empathic, and repetitive tasks in retirement house [89,90]; to transport food or medicine carts; for grasping objects [20]; for laundry collection; and, delivery services, mail delivery, warehouse logistics, trash logistics, and cleaning material logistics [91]. With the spread of these innovative technologies, the service quality can increase and, at the same time, resident and staff satisfaction improve. In this context, it is also important to personalize the human–robot interaction by endowing the robot with human-like social skills (i.e., natural language processing, user emotion estimation, etc.) [92,93].

### 3.5. Discussion: Pros and Cons of Different Technologies

**Figure 5:**
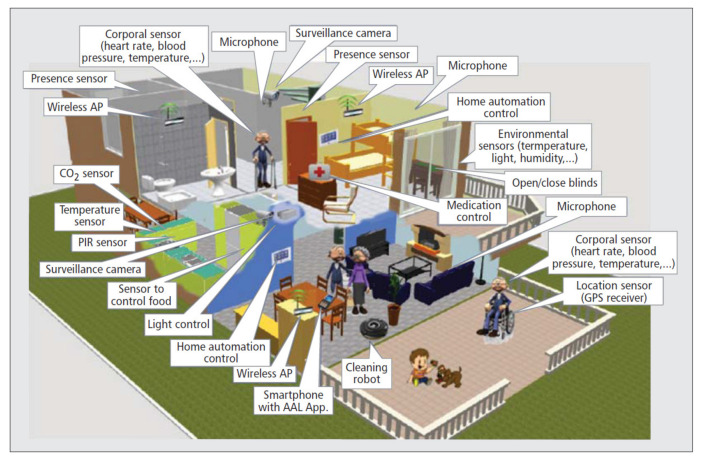
An example of AAL elderly people’s residence or house full of sensors, such as a presence sensor or temperature sensor, and actuators, such as light control, home automation control, medication control, and so on (from [94]).

The recent advancement in technological developments has made a wide variety of sensors available, even at a low cost, such as wearable rings, bracelets, smart watches, and mobile phones, for monitoring many functional parameters that are related to people’s health and activities. These sensors have to be worn and they may not be accepted by older people. Less intrusive sensors can be introduced in intelligent dresses and, together with antennas placed in the environment, can provide information for the detection of people. Other more complex sensors for monitoring vital signs are, at the moment, in an experimental phase and, although they have been shown to be effective in experimental tests, they must be miniaturized for being acceptable as wearable devices or have to be used for disease monitoring with a conscious involvement. Environmental sensors that are based on optical or microwave technologies are less invasive for people and they have been demonstrated to be robust for both activity detection and vital sign monitoring. They have to be placed properly in the environment and they may require infrastructure changes for their installation. Smart objects or sensors in furniture can be used to monitor daily life activities and interactions with objects. Additionally, in this case, some of the proposed solutions only require the addition of IoT sensors to the furniture that is already available; in other cases, it is necessary to replace objects with smart ones. In Figure 5, an example of how an elderly people’s residence or house could be structured is provided [94]: a large number of sensors could be installed at different locations of the environment, depending on the specific parameters that have to be measured and the particular events that would be monitored. It is evident that the selection of sensors among all of these possibilities depend on several factors: the costs of the sensors, their intrusiveness, their acceptability from users, the level of intervention that is required in the environments, the privacy issues that are related to the use of each sensor, and so on. Because these sensors have widely demonstrated their usefulness and potentialities, in the future they are certainly going to become of ordinary use. Thus, not only will the wearable sensors be a part of everyday life, but the environmental ones, even if they require structural interventions, will also be given in the new residences, thus making the flats and all the furniture smart.

## 4. Methodologies for Data Analysis

Many AAL systems only require data collection and the application of simple techniques for data analysis, such as threshold-based or distance-based methods. These techniques can be enough to trigger alerts when dangerous events are detected, providing interesting supports in various contexts of AAL. They only require a parameter estimation phase to be obtained by statistical analyses of the acquired data. When multiple heterogeneous sensors are used, several types of features have to be analyzed and more complex functionalities have to be recognized. The problem of feature selection becomes relevant for both reducing the cost of AAL systems by facilitating the execution of algorithms on devices with limited resources, as well as for generating reliable classification models of human activities and daily living. Several works have tested feature selection methods on large heterogeneous data comparing the results among different selections of spatio-temporal features [95,96,97].

The application of Machine Learning (ML) methodologies can solve both the problems of feature selection and data processing in order to automatically build predictive models. ML algorithms, such as Support Vector Machine (SVM), k-Nearest Neighbor (kNN), Artificial Neural Network (ANN), Naïve-Bayes (NB), Random Forest (RF), Decision Tree (DT), and Multi-Layer Perceptron (MLP), build models of each class through a training phase in a supervised learning style and then classify data in complex situations where the data are not linearly separable [98,99]. SVM and kNN are instance-based methods. They create sets of example data, and new instances of data are compared to them using a similarity measure to find the best match and make a prediction. NB is a Bayesian method that explicitly applies Bayes’ Theorem requiring the knowledge of a priori and conditional probabilities that are related to the problem under consideration. DT and RF are tree-based methods that build a model resembling a sort of decision-making diagram that is based on actual values of attributes in the data. A prediction for a given new record is obtained following the tree structure until a leaf is reached. ANN and MLP are artificial neural networks where the structures are inspired by biological neural networks. They are composed of different layers of neurons (nodes) and different activation functions at nodes for mapping the input examples to the output label of a class.

Deep Learning (DL) methodologies, such as Convolutional Neural Networks (CNN) or Long Short-Term Memory Networks (LSTMs), have been successfully applied to many application contexts in recent years, at the same time solving the problem of feature selection and model construction [100]. CNNs are mainly used in Computer Vision for visual imagery. When receiving an image as input, a CNN is able to differentiate among aspects or objects without hand-engineered filters, because CNNs have the ability to learn these filters/characteristics during training, successfully capturing spatial and temporal dependencies. LSTM networks are particularly useful for learning sequences that contain longer term patterns of unknown length, due to their ability to maintain long term memory. Furthermore, stacked LSTM networks enable the learning of higher level temporal features, for faster learning with sparser representations [101]. The large amount of data needed for the training phases and the requirement of large computational times and resources constitute the main drawback of DL methodologies.

In Table 2, the most commonly applied methodologies are resumed, together with the sensors that are used for data acquisition, the tasks, and the test sets of participants involved in the experimentation and observed activities. The data that are listed in the table have been grouped while considering the tasks mainly investigated in the development of an AAL system, i.e., fall detection, anomaly detection or abnormal behavior detection, and daily activity recognition. As can be seen in Table 2, in each cited paper experiments have been carried out over different test sets (in terms of number of subjects, subject classes, type of actions, and so on). Therefore, even when considering the same task, it is not possible to provide hints on which methodology performs better than others. Nevertheless, the literature works prove that high accuracy rates can be obtained, even when complex data must be managed.

Fall detection is an important issue that is frequently addressed in AAL literature research, as falls represent one of the main threats to the health and independence of elderly people and the main cause of injuries and hospitalization. Therefore, robust methodologies are needed for the timely detection of falls and to avoid false alarms. Various works in literature apply and compare different ML algorithms among the aforementioned ones, obtaining high accuracy rates of fall classification [37,102]. DL approaches have been also applied in this context, implementing the training phase of DL models in the cloud to learn the models of different falls, and only deploying remotely to the edge gateway the test in order to manage the limited resources and provide security and life-cycle service management [40,103].

The detection of anomalous behaviors in users’ routines is a key requirement for AAL systems, as it can provide people with remote support for chronic disease prevention. An abundance of of literature works treat this problem by providing effective solutions. Additionally, in this case, ML algorithms have found considerable applications, as they have been applied to learn the “normality” of behaviors, especially on large data recorded over different periods of time and acquired by heterogeneous sensors [70,104,105,106,107,108]. The obtained models are used to detect possible anomalies in daily behaviors, such as prolonged inactivity periods, a loss of consciousness, inability to stand up, and similar ones that require immediate medical assistance [109]. In a similar way, the models of the normal behavior can be specifically learned for each user after an initial training phase being performed on selected data and can be used to find anomalies from the normal conduct of each individual, thus reducing fake alerts from the lack of adherence to general models [110].

Different approaches for activity recognition exploit the discrimination capability of the continuous-time duration of activities and inter-occurrence times of observed events for identifying an irregularity in routine behaviors from statistical histories [111,112]. This type of approaches relies on generative stochastic models that address the recognition of sequential activities from the events, the frequency of events, the continuous duration of each activity, and the continuous time between consecutive events.

Activity recognition tasks can be also performed by studying the postures of people over long periods of time. Optical sensors, such as RGB-D cameras, allow for the extraction of three-dimensional (3-D) skeleton data that better characterize the human postures that are involved in the activities to be recognized [113]. In these cases, multiple features (such as skeleton joints coordinates, joints distances, angles among consecutive joints, and so on) have to be analyzed in spatio-temporal domains to assess different attitudes of the body. ML algorithms [114,115] or DL techniques [116] allow for the manipulation of these complex features, the extraction of significant information, and the construction of complex models that are associated with each activity. The experiments demonstrate that these approaches have good performances and allow for discerning between similar activities.

An additional important class of approaches, which has been applied in limited cases for AAL systems, are those that are based on unsupervised learning techniques. They can be applied for the clustering of large amounts of unlabelled data, grouping user behaviors, and detecting high-level variations without any prior knowledge or training phase [117,118].

### Discussion: Pros and Cons of Different Methodologies

This study has identified several clusters of options for data processing in AAL, depending on the complexity of the target. Many examples of successful applications of simple techniques for data analysis, such as threshold-based or distance-based methods, have been reported in the literature for the recognition of many elementary behaviors. Machine learning methodologies, which have been successfully applied in the contexts of pattern recognition, have been borrowed for processing data of functional and healthy parameters for the purpose of more complex behavioral analysis. Many successful examples of deep learning applications have been also reported. The main problem concerned with the use of more sophisticated machine learning is related to the amount of data that are required for the learning phase. Supervised, unsupervised, and deep approaches require large data sets to build their models. However, this large amount of data is not always available in AAL contexts, where systems must be installed and immediately operational. In many cases, it may be necessary to foresee an initial phase of data collection for the tuning of the methodologies before moving on to their actual use.

Another aspect relates to the extraction of significant features on which these systems have to work. In many cases, data may be redundant, and significant features have to be extracted. Machine learning algorithms for data classification or regression require a preliminary feature extraction phase that is fundamental in assuring good performance of the whole analysis. In many cases, data may be redundant or variegated to be easily interpreted: the task of feature extraction can be too hard to be achieved with the supervision of domain experts. However, deep learning approaches have, in some way, solved this problem, as they learn features automatically from data without the need for manual feature extraction. Conversely, they are even more data-demanding than traditional machine learning approaches.

From the analysis of the literature, it has emerged that an absolute and fair comparison among the different approaches presented in different works is not possible. Most of the approaches are applied and tested in limited contexts, such as in labs or selected use cases, and others use data extracted from publically available datasets [119,120,121]. Accordingly, the reported results are based on different experimental data; therefore, the superiority of one methodology rather than another cannot be argued. However, the previous pros and cons analysis can drive the choice amongst the vast array of approaches. In the case of simple tasks, such as binary or few-classes classification tasks, which could not be solved by simple linear discrimination (threshold operations, distance-based analysis, and so on), but involve straightforward feature engineering, classical machine learning approaches (ANN, SVM, DT, kNN, RF, and so on) are good options. On the contrary, in the case of highly unstructured data related to heterogeneous sensors and having intricate relationships between several interdependent variables, deep learning can be the best solution of complex inference, since it can discover hidden patterns, combine them, and make better decisions in an autonomous data-driven way.

## 5. Discussion

In a society where the elderly population is constantly increasing, technology can help to improve the quality of life of the elderly and extend their independent living. In recent years, AAL systems have been developed to create better living conditions for older and disabled people, and to support caregivers and medical staff with behavioral and alerting information that can be useful for prevention or timely intervention. Furthermore, when considering the recent pandemic situation, where people have been forced into isolation or require assistance, AAL systems have become essential for communication, safety, and security purposes. These systems can be used to monitor simple daily habits, quantifying, for example, movement or rest periods, verifying the meal or medicine intake, and so on, but also to highlight social components that affect psychological well-being. This is valid for both private homes or retirement homes, where many aspects of the daily activities of residents can be automatically checked, which reduces the costs and increases the monitoring capabilities.

**Figure 6:**
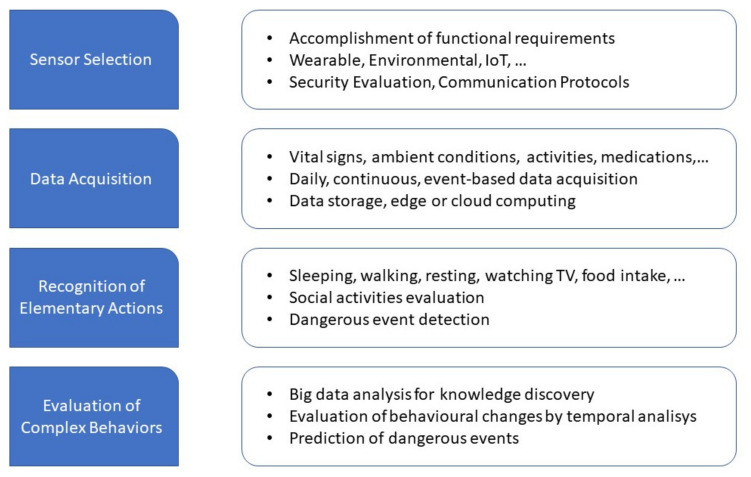
The main steps in AAL systems.

Figure 6 outlines the main steps involved in the development of an AAL system. These steps involve several strictly related choices that start from sensor selection for data acquisition up to functionalities that AAL systems intend to provide. First of all, the sensor selection depends on the costs and invasiveness levels for both people and infrastructure. The choice of the functions to be implemented also imposes constraints on the sensors to be used and the modality of data acquisition. Simple functionalities can be realized with few sensors, even at a low cost. More complex functionalities are needed in order to understand the first elementary behaviors, such as interactions with objects, daily activities, hours of rest, quality of sleep, etc., and then the resulting data have to be collected and evaluated to extract higher level knowledge, such as recognizing changes in habits. Heterogeneous sensors must be used and the data collected over a long period of time. In the same way, the methodologies for data analysis that require learning phases for model building can impose constraints on the quantity of data that has to be acquired. Subsequently, all of these aspects should be considered when complete AAL systems are developed.

The analysis of the extant literature on AAL systems has highlighted some fundamental aspects that require the attention of the scientific community.

**The need of a human-centered approach.**
  From a technological point of view, the main aspect that should be considered is certainly connected to the actual acceptability of sensors by people and, consequently, to the reliability of the acquired data not affected by behavioral conditioning due to the awareness of being observed or having behavior assessed [122]. The design of AAL systems has to consider the needs of people preferring a human-centred approach, where the end users are involved and participate in all stages of the design process [123]. A long term evaluation as part of a field trials “in the wild”, regarding functionality, acceptability, and utility of the AAL systems, in general, has only been studied in a few papers [124,125].
**The need of large amount of data from real environments.**
  Another aspect concerns the experimental tests on which the systems proposed in the literature have been evaluated. Many works propose experiments in labs, use data sets, or are applied in real contexts but with a limited period of observations. If these types of tests ensure that single functionalities work, they are rather limited for more complex behavior analysis. In order to ensure effective validity, real environment situations should be considered, where various parameters can be related to different activities that are actually performed during real scenarios of daily life.
**The need of learning the “normal” behavior of each individual.**
  The evaluation of complex behaviors, such as anomaly detection or change detection, also requires special attention. To detect abnormal changes in any monitoring system, the first step is to build a systematic model from long-term observations of normal activities. Later, the normal pattern is compared with new observations and the deviation is estimated. However, in the context of AAL systems, what behavior can be considered to be “normal”? The concept of normality is not general and it cannot be the same for different subjects. It is closely related to each individual, so it must be learned from the prolonged observation of each person. As stated before, recent technological developments have made it possible to easily collect and store a huge amount of data on people’s habits. The main point on which future research must focus is to develop methodologies that are able to process this huge amount of data and create customized models of normal behaviors. These methodologies have to select the fundamental features while discarding irrelevant information and recognize deviations from normality as soon as they arise.
**The need of adaptive systems.**
  An additional aspect is worthy of consideration: AAL systems cannot be closed, as the needs and habits of people change over time as well as the parameters to be observed. The methodologies for data analysis must consider the possibility of differently weighting or customizing some parameters rather than others dynamically. Furthermore, the models of normality must be updated in an adaptive way as people’s needs or health change.
**The evaluation of processing constraints.**
  Processing constraints also have to be considered when AAL systems must be devised. Many AAL systems have been devised to provide alarms when dangerous situations are detected. In these cases, hard real time processing of data is necessary for providing prompt interventions, but, at the same time, robust processing techniques are necessary to avoid fake alarms [126]. In some approaches, information regarding people’s behaviors has to be collected and advice provided. In these cases, soft real time processing is necessary, as data can be collected during the whole day, sent to a central server for the overall evaluation, and then the proper users can be provided with the processing results. On the contrary, long periods of observation are required when changes in habits require detection. In this case, systems have to collect data regarding all aspects of the daily life, and the results can only be evaluated when models of normal behaviors have been estimated. In these cases, offline processing can be done on a large sample of data collected in a cloud computing framework.
**The importance of data clustering.**
  The use of unsupervised approaches is very useful for clustering data and assessing the presence of common behaviors. In the following years, consistent with the predicted expanded deployment of smart objects or environmental sensors in the houses, together with the use of wearable sensors by users, large amounts of data will be available. Unsupervised approaches for data clustering will represent a useful and flexible means to analyze behaviors and extract the habits, as well as the social, behavioral, and functional aspects, of subjects for the purpose of providing medical staff with diagnostic support.
**The need of interdisciplinary competences**
  An additionally important point that is involved in the development of AAL systems is the need of interdisciplinary competences to cover all aspects related to the installation, acceptability, and functionality of an AAL system when considering the interaction levels between users and assistive technologies. Experts in technologies and methodologies for data processing, as well as doctors, geriatricians, and psychologists, must work in interdisciplinary teams to design the overall framework of robust and reliable AAL systems.

In conclusion, the next decade will likely witness a considerable spread of technologies in people’s home. Accordingly, huge sets of hard real-time data generated by residents and environment will be available. The main challenge that remains is to provide these systems with intelligence. The scientific community must focus on how to use these data, as well as on developing and incorporating intelligence through machine learning, intelligent decision making, and control methods while taking the state of residents and environments and their privacy into account.

## 6. Conclusions

The development of AAL systems has been receiving considerable attention from the scientific community in the recent years for several reasons: they can reduce the costs of daily life assistance of elderly people; they can monitor the physical and psychological wellbeing of people that live alone; and, they can detect behavioral changes that could be the sign of early stage of neuro-degenerative diseases.

The main aspects of AAL systems related to health care applications have been surveyed in this paper. This review, without any pretense of being exhaustive, proposes an overview of the main issues and choices that need to be made, while considering the possible application contexts, the available technologies, and methodologies for feature extraction and data processing. In particular, a selection of recent papers has been discussed considering the context in which they have been applied (i.e., indoors or outdoors), the target users (youngest old, middle old, oldest old people), the functional tasks that they realize (disease management, activity monitoring, change detection), the sensors used (wearable, smart objects, environmental sensors), and the methodologies applied for data processing (from simple distance methods to machine learning approaches).

As largely discussed throughout the paper, the recent development of technologies and consequent change of the habits of people, due to the introduction of these new sensors in everyday life, will make a huge quantity of data available. The main point relates to the correct usage of both technologies and data for healthy purposes. While younger generations easily and naturally utilize technologies in daily activities, elderly people could be unwilling and uninterested in using sensors, computers, or new technologies. Hence, the psychosocial factors of human–technology interaction, communication, and usage need to be considered when developing AAL systems. These factors, together with data usage and processing, are worthy of further investigations by the scientific community in order to develop increasingly complex intelligent systems for care services, remote consultation, independent living support, social participation and well-being, healthy condition monitoring, and so on.

## Figures and Tables

**Table 1:** Principal technologies applied in various contexts to create different health assistance tasks.

Sensor Type	Technology	Context	Task	Ref.
Wearable	3-axis accelerometer at user hip	Indoor	Postural Stability	[37]
Wearable	Accelerometer, gyroscope and magnetometer at the user hip	Indoor	Postural Stability	[35]
Wearable	3-axis accelerometer in a smart watch	Indoor	Postural Stability	[38]
Wearable	Smartphone at the user hip	Indoor	Fall classification	[36,43]
Wearable	IMU on people pelvis, right and left thigh	Indoor	Gait analysis	[39]
Wearable	Smartphone	Outdoor	Physical/Mental Health, Wondering Detection	[55,56]
Wearable	Wristband	Indoor	Ambient air monitoring	[44]
Wearable	RFID tag	Indoor	3D localization, Fall detection	[45,46,47]
Wearable	Wearable electrodes	Indoor	Heart rate monitoring	[52,53]
Wearable	BLE technology	Indoor	Localization	[49,50,51]
Wearable	Smart Glasses	Indoor	Vital Sign Monitoring	[54]
Wearable	Sensor Box	Outdoor	Safe Navigation	[57]
Smart objects	Sensors in appliances and furniture	Indoor	Daily life Activities, Abnormal behavior detection, Interaction with devices	[59,60,61,62,63]
Smart objects	Sensors in kitchen appliances	Indoor	Food preparation	[64,65]
Smart objects	BLE Beacons in the objects	Indoor	Interaction with devices	[66]
Smart objects	Single smart object (Cushion, wheelchair, carpet, bed)	Indoor	Specific health functionalities, sleeping posture recognition	[58,67,68,69]
Environmental	Wireless sensors in the environment	Indoor	Indoor temperature, humidity, vibration, luminosity and sound	[71,72]
Environmental	Electromagnetic Technology	Indoor	Respiration activity	[73]
Environmental	Sensor nodes in the beds	Retirement Houses	Resting time of residents	[74]
Environmental	Sensors in the environment	Indoor	Multiple People Location	[75,76,77,78]
Environmental	Multiple cameras	Indoor/Outdoor	Object detection	[79]
Environmental	Radio Frequency sensors	Indoor	Sleep monitoring, activity monitoring, changes in movement patterns, vital sign recognition	[80,81,82]
Environmental	Metasurfaces based on microwave sensors	Indoor	recognition of hand signs and vital sign recognition,	[83,84,85]
Environmental	Kinect™and Wii™	Indoor	Biomedical Sign acquisition	[2]
Environmental	Kinect™	Indoor	Activity recognition	[86]
Environmental	RFID in the wall	Indoor	Activity recognition	[70]
Environmental	Multiple Kinect	Indoor	Physical training	[87]
Environmental	Wearable and environmental sensors	Indoor	Patient monitoring and environmental parameter monitoring	[40]

**Table 2:** A list of common methodologies for data processing.

Methodology	Features	Task	Sensors	Test Set	Ref.
SVM and DT	Features extracted from filtered acceleration data samples: amplitude, time, statistics, orientation	Fall Detection	Wearable sensor: Tri-axial Accelerometer at waist	6 young adults and 2 elders performing 19 daily activities and 15 fall activities	[37]
kNN, NB, SVM and ANN	Vector magnitude of acceleration and angular velocity	Fall Detection	Wearable sensors: accelerometer, gyroscope and magnetometer at wrist and chest	17 people performing several daily activities	[102]
LSTM, GRU, SVM and kNN	Time series of accelerometer data	Fall detection	Wearable sensor: tri-axial accelerometer	23 adults and 15 elders performing several daily activities and falls	[40,103]
ANN	Spatio-Temporal Features	Anomaly detection in daily activities	Wearable sensors (accelerometer and gyroscope) and Ambient sensors	2 subjects performing 9 daily activities	[109]
Time series machine learning techniques	Time series data	Behavioral trend generation and forecasting	Sensors in the objects and Ambient sensors	4 subjects performing 6 daily activities	[110]
CT-HSMM	Stream of typed and time-stamped events	High level activities recognition	Sensors in doors and household appliances	7 activities, 28 days of observations	[111]
NB, SVM, RFs, DT, CNN, LSTM	Sensors data, activity, and context labels	Daily activity recognition	72 sensors: wearable sensors, object sensors and ambient sensors	4 subjects performing 7 daily activities	[106]
Multivariate Gaussian Distribution	Statistical features	Activity recognition	Ambient sensors: smart wall equipped with RFID sensors	4 subjects performing 12 real life daily activities	[70]
CNN	Time series	Abnormal behaviors detection	Wearable sensors	9 daily activities	[105]
RF, kNN	Spatial features	Daily activity recognition	Wearable sensor: accelerometer at chest	13 subjects performing 7 daily activities	[104]
CNN	Spatio-Temporal features	Daily activity recognition	Ambient sensors: depth camera	7 participants performing 21 sets of activities	[116]
NB, MLP, RF	Spatio-Temporal features	Daily activity recognition	Ambient sensors: RGB-D cameras	13 daily activities	[115]
ANN	Spatio-Temporal features	Daily activity recognition	Ambient sensors: depth cameras and acoustic sensors	17 subjects performing 24 daily activities	[114]
HMM	Spatio-Temporal features	Anomaly detection in daily activities	Wearable and ambient sensors	10 subjects performing daily activities over 3 months of observation	[112]
LSTM, RNN	Individual sensor events or group of sensor events in various time periods	Changes in behavioral patterns	IoT sensors: sensors in objects and furniture	6 elderly people observed at home over a period from 1.5 to 4 months	[108]
Unsupervised Learning	Spatio-Temporal features	Mild Cognitive Impairment Detection	Ambient sensors: motion sensor and door sensor	10 elderly people	[117]
Unsupervised Learning	Temporal features connected to temporal cluster of sensor events	Behavioral change detection	Ambient sensors: PIR sensors	Selection of data from Aruba data set: 28-day observation period	[118]

## Data Availability

Not Applicable.

## Abbreviations

The following abbreviations are used in this manuscript:

AAL	Actice and Assistive Living
IoT	Internet of Things
ICT	Information and Communications Technology
IMU	Inertial Measurement Units
RFID	Radio Frequency IDentification
BLE	Bluetooth Low Energy
GPS	Global Positioning Systems
HR	Heart Rate
BCG	Ballistocardiography
SCG	Seismocardiography
ML	Machine Learning
SVM	Support Vector Machine
kNN	K-Nearest Neighbor
ANN	Artificial Neural Network
NB	Naïve-Bayes
RF	Random Forest
DT	Decision Tree
MLP	Multi-Layer Percepron
DL	Deep Learning
CNN	Convolutional Neural Network
LSTM	Long Short-Term Memory Network
RNN	Recurrent Neural Network
GRU	Gated Recurrent Unit
HMM	Hidden Markov Model
CT-HSMM	Continuous-Time Hidden Semi-Markov Model
